# Brain Inflammation, Blood Brain Barrier dysfunction and Neuronal Synaptophysin Decrease after Inhalation Exposure to Titanium Dioxide Nano-aerosol in Aging Rats

**DOI:** 10.1038/s41598-017-12404-5

**Published:** 2017-09-22

**Authors:** Clémence Disdier, Monique Chalansonnet, François Gagnaire, Laurent Gaté, Frédéric Cosnier, Jérôme Devoy, Wadad Saba, Amie K. Lund, Emilie Brun, Aloïse Mabondzo

**Affiliations:** 1 Service de Pharmacologie et d’Immunoanalyse, UMR 0496, CEA, Université Paris-Saclay, F-91191 Gif-sur-Yvette, France; 2 INRS, Département Toxicologie et Biométrologie, Rue du Morvan, CS 60027, 54519 Vandœuvre Cedex, France; 3 Inserm, CEA, Université Paris-Saclay, UMR 1023 - ERL 9218 CNRS, IMIV, Orsay, F-91406 France; 4 Department of Biological Sciences, Advanced Environmental Research Institute, University of North Texas, Denton, TX USA; 5 Laboratoire de Chimie Physique, UMR CNRS 8000 Université Paris-Saclay 91405, Orsay, France

## Abstract

Notwithstanding potential neurotoxicity of inhaled titanium dioxide nanoparticles (TiO_2_ NPs), the toxicokinetics and consequences on blood-brain barrier (BBB) function remain poorly characterized. To improve risk assessment, we need to evaluate the impact on BBB under realistic environmental conditions and take into account vulnerability status such as age. 12–13 week and 19-month-old male rats were exposed by inhalation to 10 mg/m^3^ of TiO_2_ nano-aerosol (6 hrs/day, 5 day/week, for 4 weeks). We showed an age-dependent modulation of BBB integrity parameters suggesting increased BBB permeability in aging rats. This alteration was associated with a significant increase of cytokines/chemokines in the brain, including interleukin-1β, interferon-γ, and fractalkine as well as a decreased expression of synaptophysin, a neuronal activity marker. These observations, in absence of detectable titanium in the brain suggest that CNS-related effects are mediated by systemic-pathway. Moreover, observations in terms of BBB permeability and brain inflammation underline age susceptibility. Even if TiO_2_ NPs were not evidenced in the brain, we observed an association between the exposure to TiO_2_ NPs and the dysregulation of BBB physiology associated with neuroinflammation and decreased expression of neuronal activity marker, which was further exacerbated in the brain of aged animal’s.

## Introduction

Due to their remarkable properties, nanoparticles (NPs) could potentially be used in a large array of applications, from electronics to medicine. Nanomaterials are currently being used in the production or manufacturing processes of a number of commercial products^1^. In this context, there are increasing concerns regarding the potential adverse effects of NPs on human health. Among the wide variety of nanomaterials, titanium dioxide NPs (TiO_2_ NPs) are produced in a large industrial scale and can be found in commercial products such as paints, food additives^2^, cosmetics^3^, and environmental decontamination systems^4^. Published data from studies in rodents show that TiO_2_ NPs can cross the epithelial barriers (nasal, bronchial, alveolar, gastro- intestinal) and enter the blood circulation from the respiratory and the gastro-intestinal tracts^5–10^. After inhalation exposure, there is larger concern about translocation of NPs into the brain, either directly *via* the olfactory pathway or indirectly across the blood brain barrier (BBB). Indeed, ambient air particles and NPs have been previously demonstrated to translocate into the brain after inhalation, and thus may potentially influence the central nervous system (CNS). For example, translocation of 36 nm ^13^C particles was observed in the olfactory bulb of rats, most likely originating from entry *via* the olfactory mucosa of the nose^11^. Brain translocation of gold NPs were also observed in a rat exposure model^12^, and 15–20 nm iridium NPs could be detected in the rat brain up to 6 months after inhalation exposure^13,14^. Despite these observations, the effects of NPs on the CNS under realistic environmental or occupational exposure conditions have not yet been fully characterized. The few reports, to date, reveal increased oxidative stress, activated inflammatory pathways, modulation of neurotransmitter levels in the brain, and impairment of spatial recognition associated with the presence of Ti in the brain^15–22^. However, the exposure protocols used in those studies suffer from a lack of representability to draw appropriate conclusions about potential neurotoxicity of TiO_2_ NPs. Moreover, none of these studies looked at potential BBB dysfunctions, which is known to promote neurotoxicity and is recognized as the key mechanism in many CNS-related diseases. Furthermore, recent evidence from several studies suggests that exposure to air pollution containing fine particles can increase the risk of fatal stroke, cause cerebrovascular damage and neuroinflammation that may trigger neurodegenerative diseases such as Alzheimer’s and Parkinson’s diseases^23–26^. Altogether, these observations warrant further research to investigate the potential effects of TiO_2_ NPs on cerebrovascular function.

If consequences of NP exposure on cerebrovascular functions are explored, then aging should also be considered, since increased BBB permeability has been demonstrated in the aging brain, associated with elevations in inflammation (*via* redox imbalance) that could ultimately result in cytotoxic activation of glial cells and neurodegeneration^27–29^. Aging is likely a critical vulnerability parameter to consider with NPs exposures, and which has been overlooked in many aspects of nanotoxicology research so far.

We have previously reported Ti tissue distribution after subacute inhalation of TiO_2_ nano-aerosol in adult and aging rats up to 180 days post-exposure, which revealed evidence of titanium persistence in lung and translocation to secondary organs such as liver and spleen^30^. In this previous work, we exposed young adult Fischer rats (12–13 weeks) and aging rats (19 months) to 10 mg/m^3^ of a well characterized TiO_2_ nano-aerosol for 6 hrs/d, 5 d/wk for a period of 28 days, dose which corresponds to the 8 hours time-weighted average French Occupational Exposure Limit value for particles without known significant toxicity^31^. Moreover, inhalation of 10 mg/m^3^ of ultrafine (<100 nm) TiO_2_ dusts was the lowest concentration associated with cancer of the respiratory tract in rats^32^. In this paper, we now focus on the consequences of TiO_2_ distribution on the CNS and particularly on the BBB functions as well as on neuronal function at the end of the inhalation period up to 28 days.

The two hypothesis underpinning the current study are: (1) inhalation exposure to a TiO_2_ nano-aerosol could result in BBB permeability alteration and subsequent brain inflammation, and (2) aged rats could be more susceptible to CNS dysfunctions following nano-aerosol exposure. To investigate these topics, *in vivo* BBB permeability was evaluated with a marker of paracellular transport and tight junction protein expression. Total brains were analyzed for neuroinflammation and a neuronal activity biomarker, after a recovery period of 3 and 28 days. A minimum of 3 days recovery was maintained to ensure appropriate conclusions about potential effect on the CNS due to TiO_2_ nano-aerosol.

## Results

### Translocation of titanium to the central nervous system

**Figure 1:**
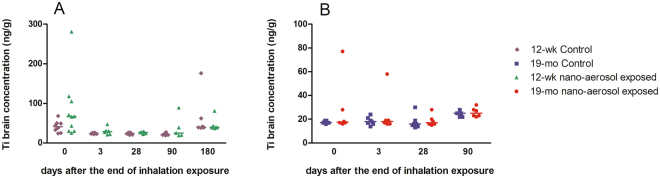
Titanium quantification in brains of young adult rats (**A**) and aging rats (**B**) of exposed and control animals by inductively coupled plasma mass spectrometry (ICP-MS) at 3 days and 28 days after the end of the inhalation exposure. Results represents the median of n = 5 to 8 animals. Statistical comparison between treated and control groups was by two way ANOVA after Box-Cox transformation on Ti concentration variable and Bonferroni post hoc test.

At the end of the 28-day inhalation period, animals were held for a recovery period of 3 or 28days. At each time point (n = 6–12 rats per time point), brains were collected for titanium (Ti) analysis by inductively coupled plasma mass spectrometry (ICP-MS), as described previously^33^. Titanium concentration in the brains of young adults and aging rats after subacute inhalation exposure to TiO_2_ nano-aerosol is reported in Fig. 1. In both age groups, Ti analysis revealed lack of detectable translocation to the brain.

### BBB integrity assessment

**Figure 2:**
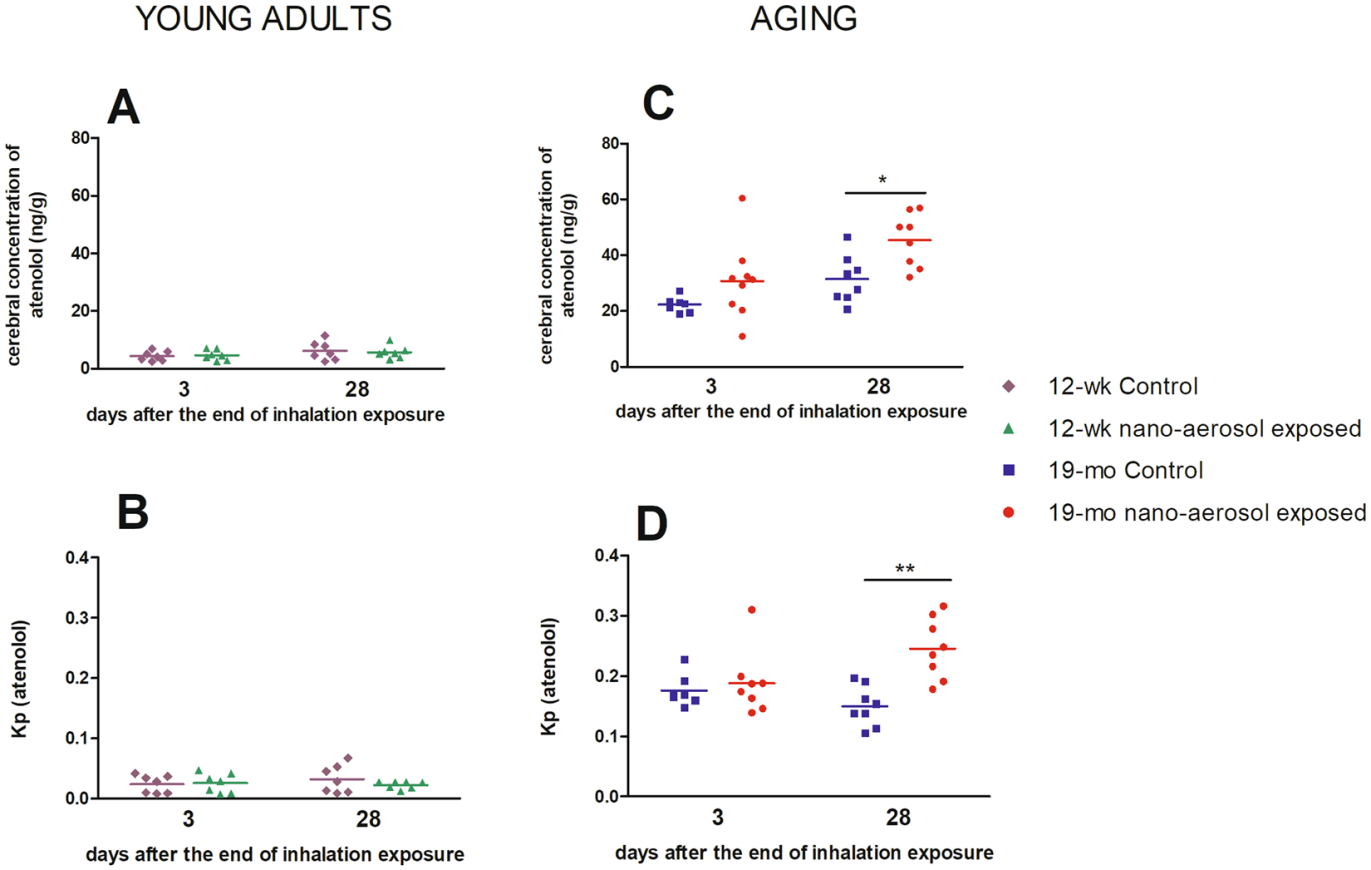
BBB permeability assessment. The permeability was estimated by the atenolol cerebral concentration and the ratio between atenolol in the brain and plasma (partition coefficient or Kp) at 3 days and 28 days after the end of the inhalation exposure in young adults (**A** and **B**) and aging groups (**C** and **D**). Each data point represents one animal with median of n = 7 to 9 rats. *P < 0.05; **P < 0.01 compared to corresponding control.

The BBB is a physical barrier that limits the paracellular passage of xenobiotics in the brain. This limitation is explained by the presence of protein junctions between neighboring brain endothelial cells (BECs). To assess indirect effect of Ti on BBB integrity, the expression of tight junction (TJ) protein Claudin 5, as well as the partition coefficient (Kp) of atenolol (a known marker of paracellular transport across the BBB), were determined. Under normal physiological conditions, atenolol does not cross the BBB, and thus concentrations and cerebral Kp should remain low. Conversely, an increase in cerebral Kp of atenolol suggests a permeability alteration of the BBB, allowing for atenolol to cross into the brain and accumulate. The average cerebral atenolol Kp in the young control rats was lower than in the aged control rat brains, attesting to the increase in BBB permeability with aging (0.16 ± 0.02 *vs* 0.03 ± 0.01, respectively) (Fig. 2B and D). In the young adults group, cerebral atenolol concentrations (Fig. 2A) and Kp (Fig. 2B) between the control and exposed animals remained unchanged, suggesting a lack of BBB permeability modulation. In contrast, results from the aged rat brains showed that atenolol Kp remained unchanged at day 3, but significantly increased at day 28 post TiO_2_ nano-aerosol-exposure (Fig. 2D; 0.2 ± 0.05 in exposed *vs* 0.2 ± 0.03 in controls).

**Figure 3:**
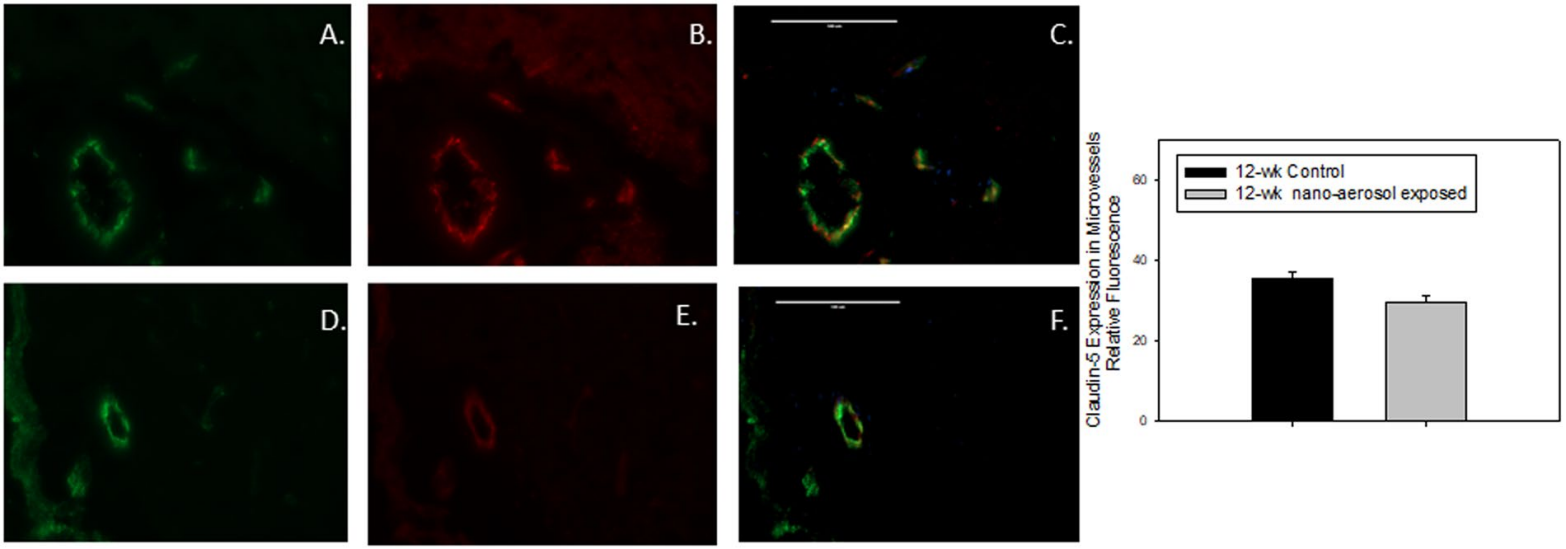
Representative expression of (**A**,**D**) von Willebrand factor, green fluorescence; (**B**,**E**) claudin-5, red fluorescence; (**C**,**F**) vessel-specific claudin-5, yellow overlay in midbrain microvessels (<100 µm in diameter) of (young) 12–13 wk control (**A**,**B**,**C**) or nano-aerosol-exposed (**D**,**E**,**F**) rats at 28 days after the end of exposure. Graph shows quantification of fluorescence shown in panels C and F. A minimum of 3 locations on each section (2 sections per slide), 3 slides and n = 3 per group were analyzed. Scale bar = 100 µm. *p < 0.050 compared to control rat microvessels.

**Figure 4:**
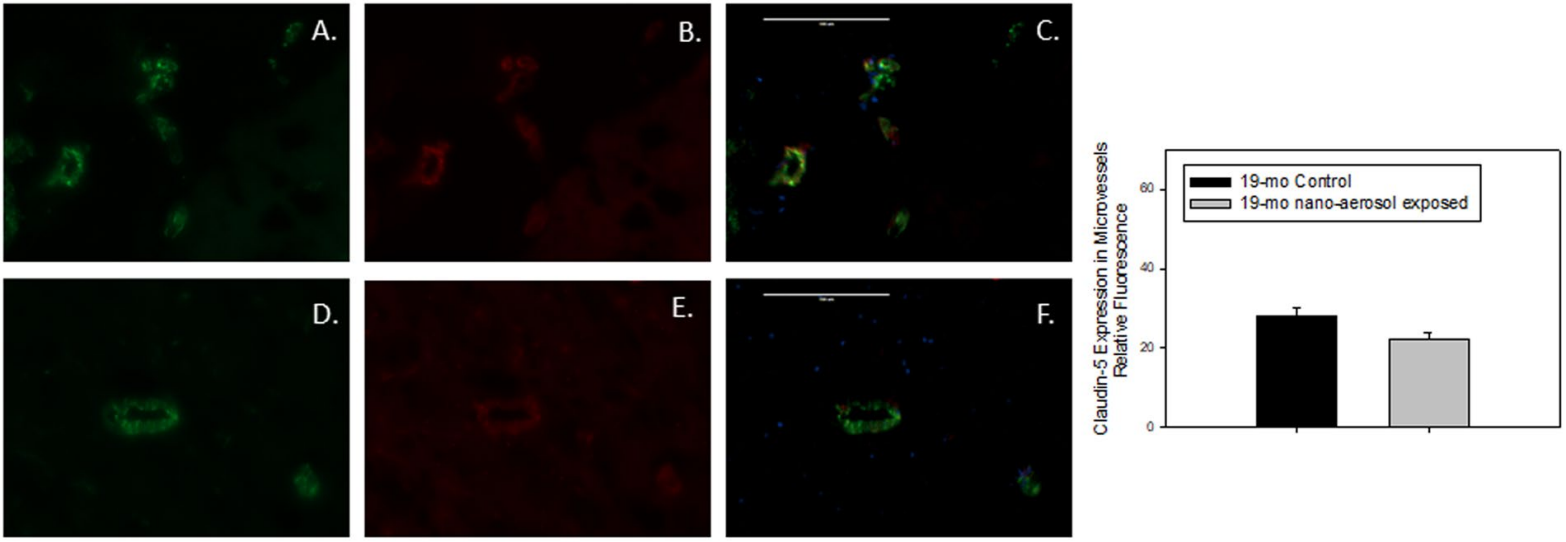
Representative expression of (**A**,**D**) von Willebrand factor, green fluorescence; (**B**,**E**) claudin-5, red fluorescence; (**C**,**F**) vessel-specific claudin-5, yellow overlay in midbrain microvessels (<100 µm in diameter) of (aged) 19 mo control (**A**,**B**,**C**) or nano-aerosol-exposed (**D**,**E**,**F**) rats at 28 days after the end of exposure. Graph shows quantification of fluorescence shown in panels C and F. A minimum of 3 locations on each section (2 sections per slide), 3 slides and n = 3 per group were analyzed. Scale bar = 100 µm. *p < 0.050 compared to control rat microvessels.

To characterize more precisely the permeability modulations at the BBB level observed after 28 days of recovery, brains from control and exposed animals were sampled and a﻿ TJ protein expression was assessed by double-immunofluorescence (Figs 3 and 4). Expression of claudin-5, one of the primary proteins expressed in TJ intercellular connections between adjacent BECs that contribute to BBB structural integrity, was down-regulated at 28 days after the end of the TiO_2_ nano-aerosol inhalation period in both the young and aged rat brains, compared to control animals (quantification graphs represented in Figs 3 and 4). The decrease of TJ protein expression in the cerebrum of the TiO_2_ nano-aerosol-exposed aged rat correlates with an increased alteration of BBB integrity (Figs 2C,D and 4). Interestingly, the claudin-5 expression decrease observed in the microvasculature of the young rat brains (Fig. 3) was not accompanied with an alteration of BBB permeability, as shown in Fig. 2A,B.

### Neuroinflammation assessment

**Figure 5:**
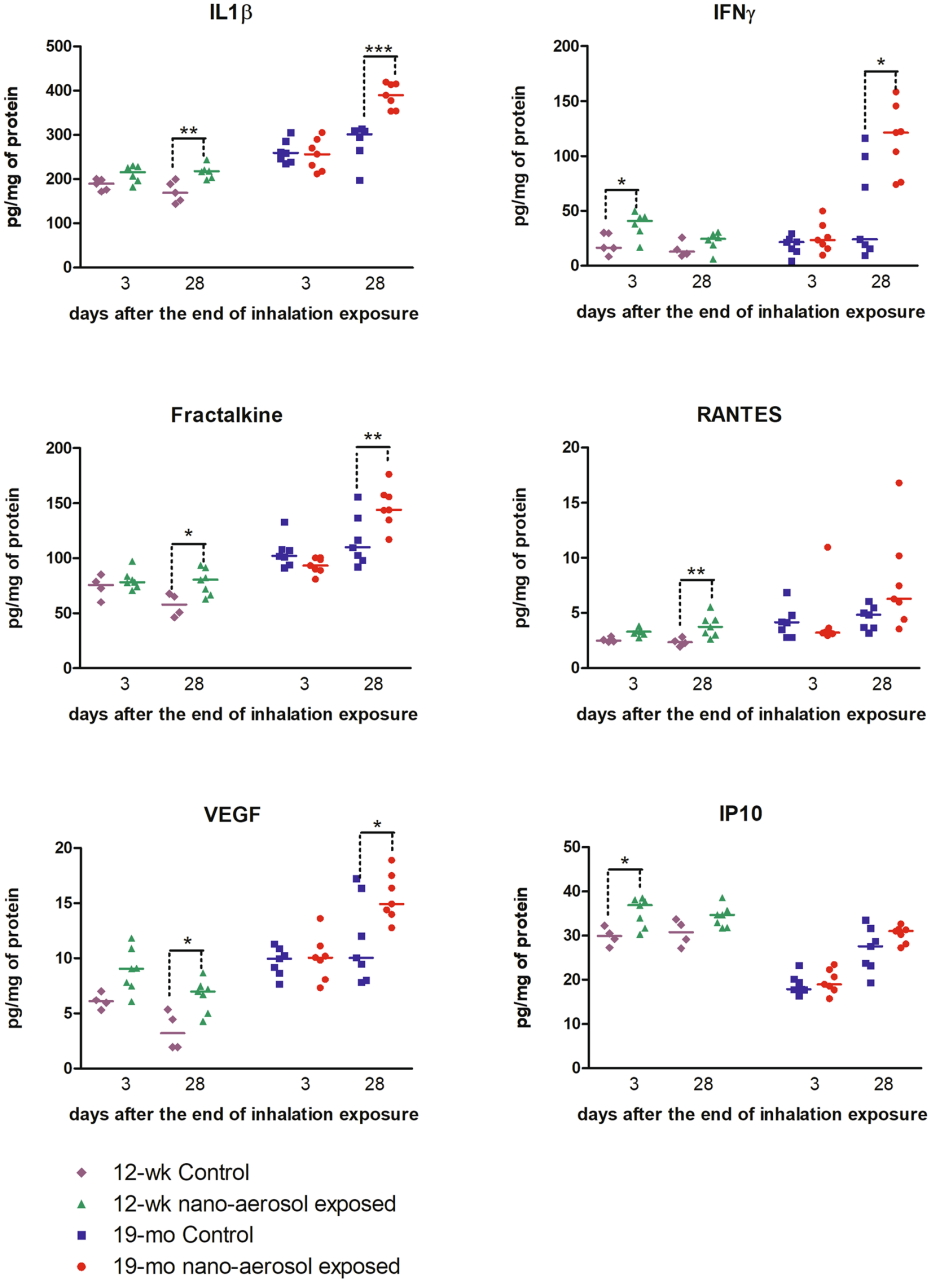
Detection of 6 inflammatory markers in young adults and aging rat cerebral tissues (cerebrum + cerebellum) after subacute inhalation exposure to TiO_2_ nano-aerosol. Measurement was done in cerebral tissues extracts collected 3 or 28 days after the end of the inhalation exposure by multiplexing approach. Each data point represents one animal with medians of 4 to 7 animals. *P < 0.05; **P < 0.01 compared to corresponding controls.

**Figure 6:**
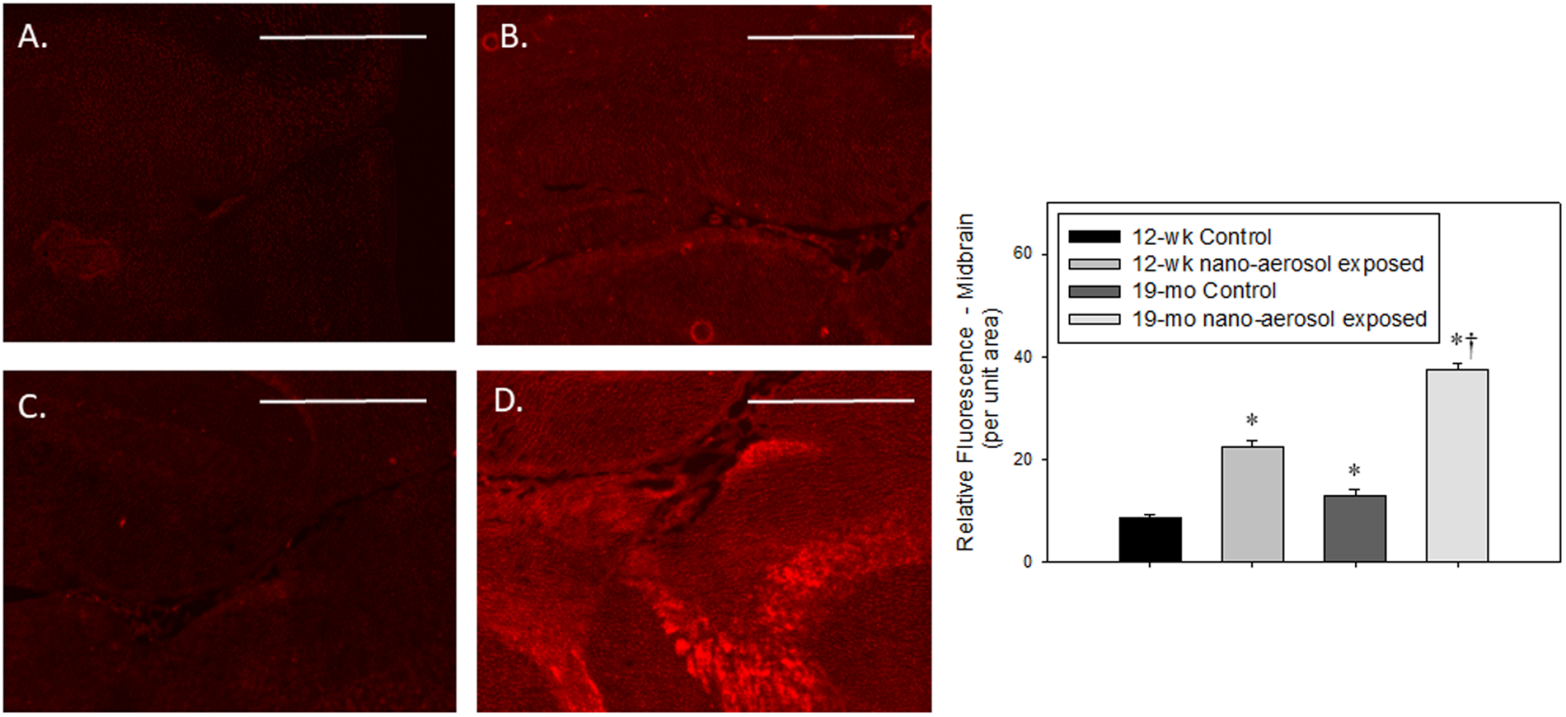
IL-1β expression (red fluorescence) in the midbrain of (**A**) 12–13 wk control, (**B**) 12–13 wk nano-aerosol-exposed; (**C**) 19 mo control; and (**D**) 19 mo nano-aerosol-exposed rats at 28 days after the end of exposure. A minimum of 3 locations on each section (2 sections per slide), 3 slides and n = 3 per group were analyzed. Relative fluorescence per unit area is represented in graph shown. *p < 0.050 compared to 12–13 wk control; ^†^p < 0.050 compared to 19 mo control. Scale bar = 1000 µm.

**Figure 7:**
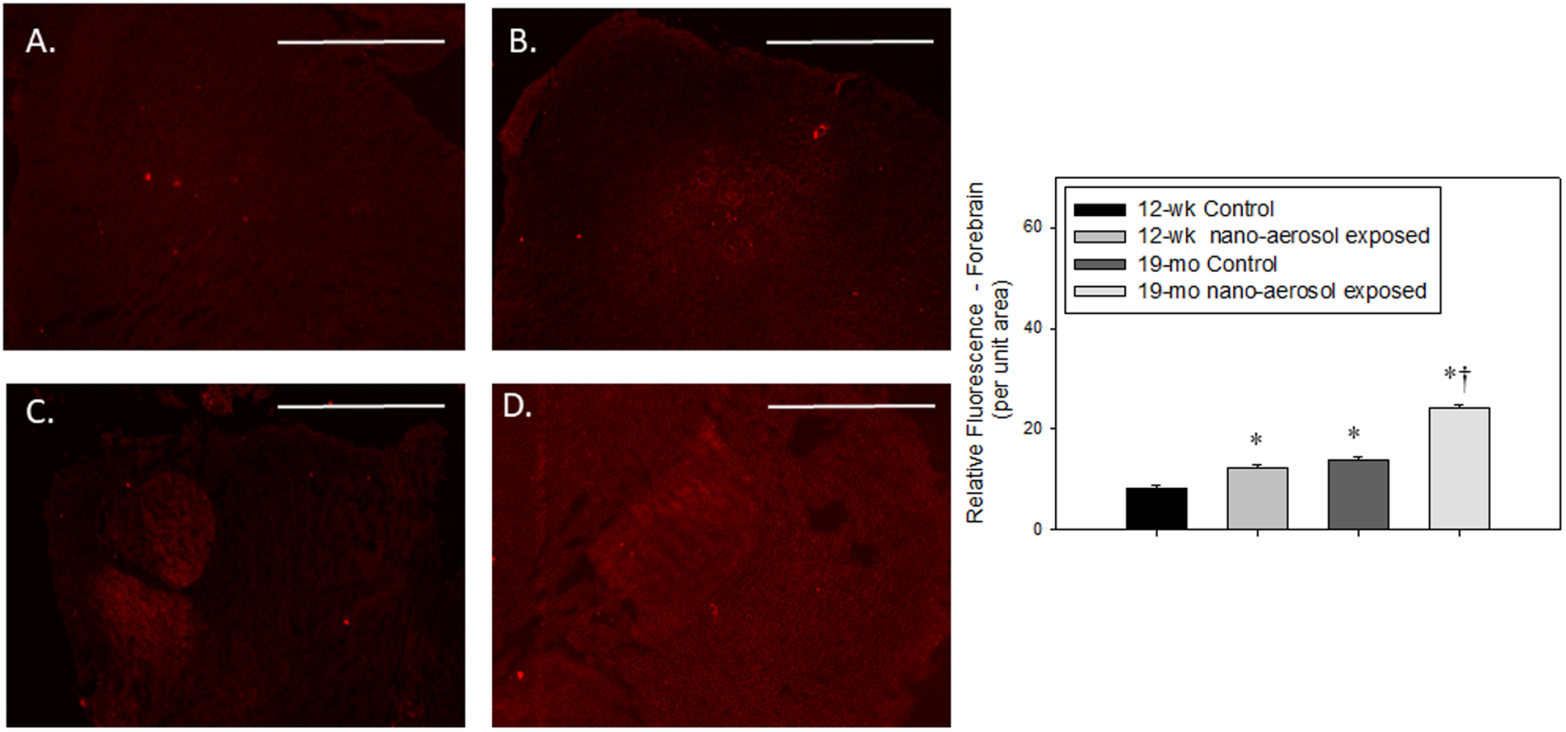
IL-1β expression (red fluorescence) in the forebrain of 12–13 wk control (**A**), 12–13 wk nano-aerosol-exposed (**B**); 19 mo control (**C**); and 19 mo nano-aerosol-exposed rats (**D**) 28 days after the end of exposure. A minimum of 3 locations on each section (2 sections per slide), 3 slides and n = 3 per group were analyzed. Relative fluorescence per unit area is represented in graph shown. *p < 0.050 compared to 12–13 wk control; ^†^p < 0.050 compared to 19 mo control. Scale bar = 1000 µm.

To determine whether TiO_2_ nano-aerosol exposure results in neuroinflammation process that can promote increased BBB permeability, we quantified cytokines and chemokines in the serum, olfactory bulbs, and cerebral tissues (cerebrum and cerebellum) from control and exposed rats of both age groups. Interestingly, assays in the olfactory bulb homogenates did not reveal any modulation in levels of inflammatory markers (data not shown). In both age groups, we noted a significant increase in the expression of several pro-inflammatory cytokines and chemokines in the cerebral tissues extracts (Fig. 5). Interleukin-1β (IL-1β) was found to be significantly upregulated 28 days after the end of the inhalation exposure in both age groups. Furthermore, the increase in IL-1β expression in the cerebrum of TiO_2_ exposed animals (both young and aged), compared to controls, was confirmed by immunofluorescence staining (Figs 6 and 7). In particular, we observed an increase in IL-1β localization in the midbrain (Fig. 6B and D) and forebrain (Fig. 7B and D) in TiO_2_ exposed brains compared to the respective age-matched controls (Fig. 6A and B and Fig. 7A and B). We observed an increase in VEGF (Vascular Endothelial Growth Factor) and fractalkine at 28 days post-inhalation in both age groups. IFNγ (Interferon-gamma), IP-10 (IFN-gamma-inducible protein 10) were also increased at 3 days and RANTES (Regulated on Activation, Normal T cell Expressed and Secreted) at 28 days after the end of the inhalation period in young adult group brains.

Cytokine and chemokine concentrations were also assessed in the serum from controls and treated rats in both age groups. Among the 11 cytokines and chemokines targeted, we could quantify only 5 analytes. These non-exhaustive assays did not demonstrate any upregulation of pro-inflammatory markers in the blood of exposed animals (data not shown).

### Impact on neurons activity

**Figure 8:**
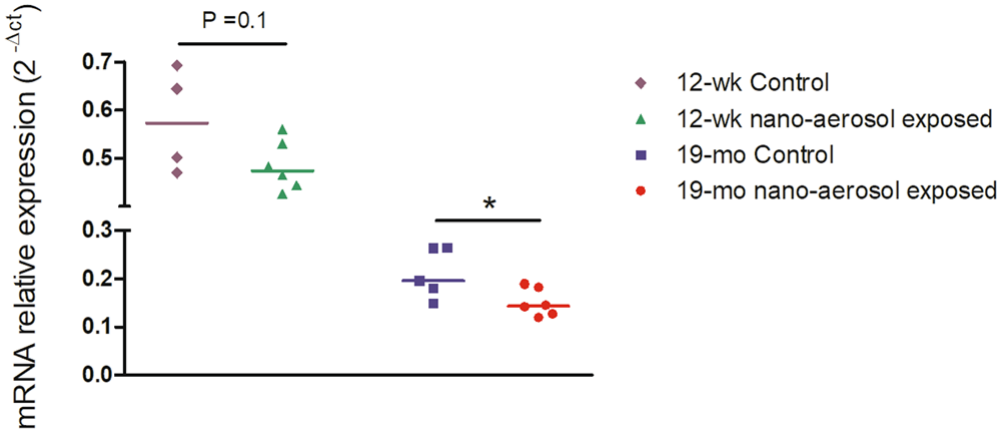
mRNA expression of synaptophysin in brain parenchyma at 28 days after the end subacute inhalation exposure to TiO_2_ nano-aerosol of young adults and aging rats. Each data point represents one animal with median of n = 4 to 6 animals and RT-qPCR was performed in duplicate for each single preparation of brain tissues. Statistical comparison was performed by Student’s t-test, *P < 0.05.

**Figure 9:**
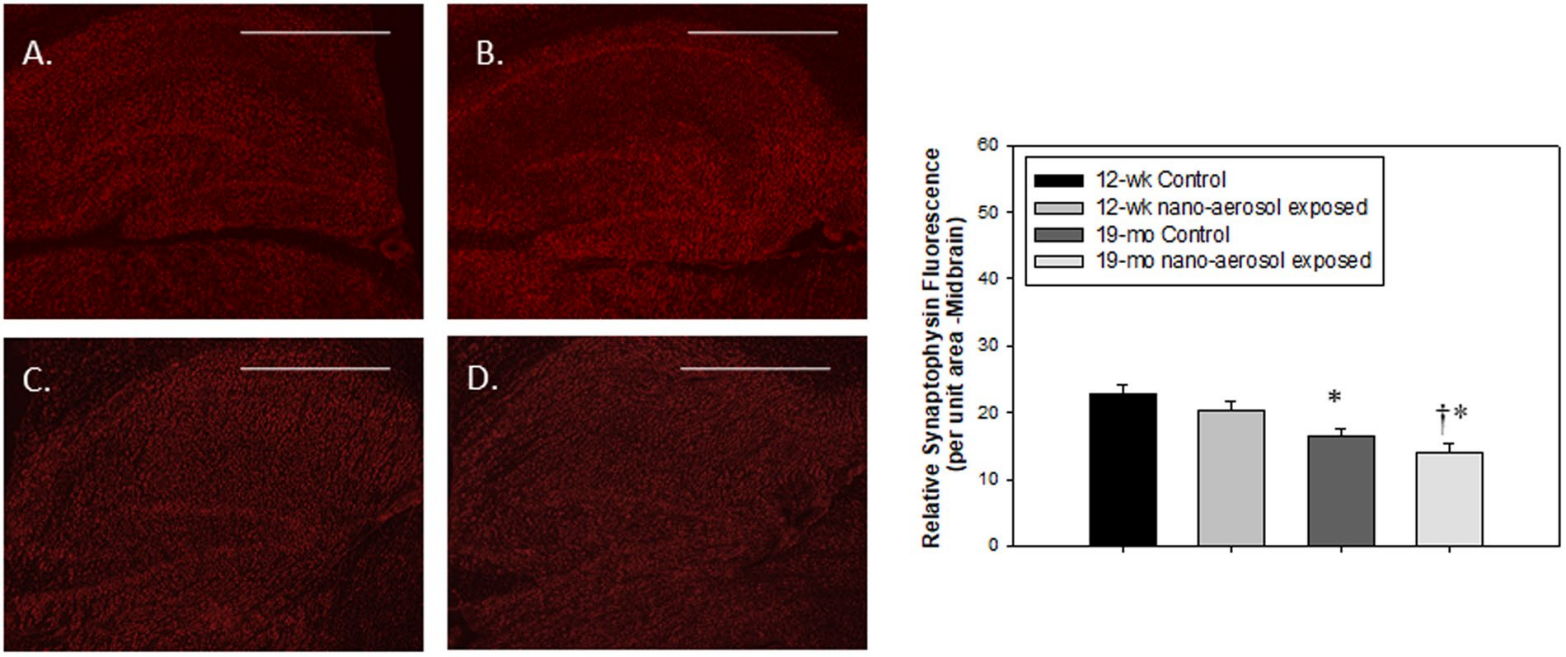
Synaptophysin expression (red fluorescence) from the midbrain region of (**A**) 12–13 wk control; (**B**) 12–13 wk nano-aerosol-exposed; (**C**) 19 mo control; and (**D**) 19 mo nano-aerosol-exposed rats at 28 days after the end of exposure. A minimum of 3 locations on each section (2 sections per slide), 2 slides and n = 3 per group were analyzed for total fluorescence. Relative fluorescence per unit area is represented in the graph shown. *P < 0.050 compared to 12–13 wk control; ^†^p < 0.050 compared to 19 mo control. Scale bar = 1000 µm.

To evaluate neurons activity, the cells constituting brain parenchyma (neurons and glial cells principally) were isolated and the transcriptional expression of synaptophysin, a neuron activity marker was evaluated 28 days after the end of the inhalation exposure. Synaptophysin is a transmembrane glycoprotein of synaptic vesicles that is essential for neurotransmission. Data depicted on Figs 8 and 9 show first that aging results in decreased basal synaptophysin mRNA (relative mRNA expression of 0.6 ± 0.1 in young adults control group *versus* 0.2 ± 0.05 in the aging control group) and protein expression, respectively. Second, a slight non-significant decrease in synaptophysin mRNA in the brain of young adult rats was observed after TiO_2_ nano-aerosol exposure (Figs 8 and 9A vs. 9B). By contrast, a decrease of synaptophysin mRNA expression in the aging brains was reported between unexposed and exposed animals (P = 0.04), which was confirmed at the protein level by immunofluorescence (Fig. 9C vs. 9D, P = 0.004). This adds to the modification of BBB permeability and the strong neuroinflammation response in the aging brain to complete the description of the cerebrovascular unit functions.

## Discussion and Conclusions

Translocation of NPs to the CNS is a key event to understand potential neurotoxicity. In case of blood distribution, NPs have to interact with the gatekeeper BBB to gain access to the brain parenchyma. After inhalation or intranasal administration, another route of entry to the brain has been highlighted: the olfactory pathway. This path connects directly the nasal mucosa and the brain^34^ and thus allows to bypass the BBB. After crossing the epithelium of the nasal mucosa, NPs can use nerve pathways to reach deep regions of the brain^35^. In this study, the nanostructured aerosol was composed of agglomerates of TiO_2_ NPs (primary size about 21.5 nm). Since aerosols found in workplace resulting from nano-powder handling and processing are composed of aggregates and/or agglomerates, the generated aerosol could be considered representative of an occupational exposure^36^. Moreover, it is worth noticing that even agglomerated NPs present a greater reactive surface area than bulk material; this may promote their capabilities to interact with biological system and their adverse effects^37^. After subacute inhalation exposure to a well characterized TiO_2_ nano-aerosol^38^, we described a lack of detectable CNS translocation in young adult and aging rats^30^. This suggests that TiO_2_ nano-aerosol did not gain access in quantifiable amount to the CNS either across the BBB or through axonal translocation from the nasal mucosa. The lack of translocation across the BBB from the blood compartment is in good agreement with our previous observations showing absence of titanium in brain parenchyma after i.v. administration of TiO_2_ NPs^39^. Moreover, given the agglomeration state of our nano-aerosol, one should have thought that small NPs aggregates could deposit in the nose and thus follow nerve pathway to the brain. This nerve pathway was hypothesized in Wang *et al*. who reported the presence of Ti in the olfactory bulb, hippocampus, cerebral cortex, and cerebellum associated with inflammation after nasal instillation in a mouse model (500 µg/mouse; TiO_2_ 80 or 150 nm rutile; daily for 30 days)^15,16^. Our results differ greatly from those of Wang *et al*. but the route of exposure, the concentrations used combined with a high dose delivery rate may have increased, in their case, NP translocation *via* the olfactory nerve pathway through non-physiological mechanisms. This may explain the differences between both studies. However, as we did not regionalize brain structures for titanium analysis, we cannot totally exclude that small amount of titanium in olfactory bulb or other areas may have gone unnoticed. Analysis of isolated brain region was unfortunately not technically possible due to the quantity of tissues needed to reach the limit of quantification of the ICP-MS method. Recently, a study by Pujalte *et al*. also failed to demonstrate significant translocation of TiO_2_ NPs (anatase 20 nm) after 6 h nose-only inhalation exposure to 15 mg/m^3^ ^40^. In this study, they detected a very slight increase suggesting trace of Ti in the olfactory bulb. To test the hypothesis of Ti trace in the olfactory bulb after translocation along the olfactory nerve, we analyzed cytokines and chemokines expressions in this specific area. Moreover, after nasal instillation of 65.3 µg, 653.8 µg or 1.3 mg of our NPs per rat during 10 days we didn’t detect titanium in olfactory bulbs of exposed rats 24 hours after instillation (data not shown). Altogether, our observations in the olfactory bulb after nasal instillation and inhalation suggest that it is unlikely that the TiO_2_ NPs tested can gain access in quantifiable amount to the CNS *via* the olfactory pathway. So, in our study, no difference in Ti concentrations were detected between control and exposed animals, but it was worth investigating potential BBB dysfunctions as we previously demonstrated that after i.v. administration, the Ti biopersistence in peripheral organs was associated with dysregulation of the BBB and neuroinflammation^39^. In the same manner, after inhalation of TiO_2_ nanoaerosol, we described biopersistence of titanium mainly in the lungs but also in liver and spleen where they could promote systemic health effects.

The first endpoint we explored was BBB integrity. Indeed, the BBB is responsible for a strict control of the exchanges between the blood and brain compartments, providing a unique protection against many xenobiotics and pathogens due to its restricted and controlled permeability. In this study, we report an increased BBB permeability in exposed aging rats, characterized by increases of atenolol cerebral concentration and Kp compared to corresponding controls. We also noticed an increased atenolol Kp between young adults and aging control groups. This age-related difference in BBB permeability emphasizes the structural alteration of the BBB with aging as already reported, in particular the alteration in tight junction protein expression^28,41,42^. Altogether, BBB permeability evaluation suggests that TiO_2_ nano-aerosol exposure results in exacerbation of BBB integrity loss that could be associated with age-related alterations. The decreased expression of claudin-5 also underscores age-related vulnerability and differences in response of the BBB to TiO_2_ nano-aerosol exposure. The difference in toxicological sensitivity between old and young adult rats may be caused by the BBB impairment of repair mechanisms or adaptive response to stress in the aging brain^28,43^. These findings further underscore the need of investigation of NPs toxicity in “at risk” populations, such as the elderly.

It is known that BBB integrity can be affected by pro-inflammatory mediators (cytokines and chemokines)^39,44,45^. These increased expressions are an early event that can lead in BBB impairment. These mediators can initiate changes in permeability and adhesion properties of BECs that allow immune cells to infiltrate the CNS. As we previously described increase expressions of pro-inflammatory mediators at the BBB or in the brain parenchyma after i.v. administration^46^ and in an *in vitro* model of BBB after direct exposure to the same NPs^47^, we were concern about potential neuro-inflammation as a mechanism of BBB physiology alteration. In this work, we quantified cytokines and chemokines in cerebral extracts and observed marked differences between control and exposed animals for IL-1β, VEGF, IP-10, fractalkine, and RANTES. IL-1β is a key mediator of neuroinflammation, particularly in the context of neurodegenerative diseases such as Alzheimer disease or Parkinson’s disease^48^. Both aging and inflammation in the brain are known to mediate memory deficits *via* IL-1β^49,50^. A systemic injection of IL-1β in mice leads to a reduction of the tight junction protein occludin by proteolysis and a subsequent breakdown of BBB permeability^51^. Our results show that in aged rat brains, TiO_2_ nano-aerosol exposure results in an even further increase in IL-1β expression compared within young rat brains, which could be involved in the BBB disruption. Among other detected markers, VEGF is responsible for regulating microvascular permeability to blood plasma proteins. Therefore, the reported increase in BBB permeability in the aged group may be due in part to the increased expression of VEGFand/or Fractalkine whichregulates trafficking and adhesion of immune cells at endothelium^52^. As a soluble chemokine, it acts as a chemoattractant for T-cells and monocytes, and promotes leukocytes adhesion. Finally, IP-10 and RANTES up-regulation underscore the inflamed environment in the brain of exposed animals^53^. Thus, up-regulation of cytokines/chemokines could mediate the increase in BBB permeability observed in our NP-exposed animals. The increased levels of adhesion molecules in the brain of TiO_2_ NP-exposed rats could suggest a possible infiltration of immune cells in the brain, which could also exacerbate BBB disruption. However, further studies are needed to determine whether immune cells infiltration is increased in the CNS after NP exposure.

Importantly, this increase in inflammatory marker expression and signaling occurs in the brain even in the absence of detectable Ti accumulation. As we did not observe increased expression of these pro-inflammatory markers present in the olfactory bulb, this highly suggests that increased inflammatory signaling may be originating at the BBB. This premise is confirmed by a similar induction of cerebral inflammation, in our previously reported study, following i.v. administration of the same TiO_2_ NPs to rats^39^. In this previous study, we described a biopersistence in the liver, spleen and lungs associated with BBB impairment and neuroinflammation up to one month after a single i.v. administration. Drawing on the outcomes showing dysfunction of the BBB and neurovascular inflammation in the absence of titanium in the brain, the hypothesis of circulating mediators originating from organs accumulating TiO_2_ NPs was assumed. We previously demonstrated that circulating mediators that may be released by organs bioaccumulating Ti can lead to neurovascular inflammation and BBB physiology alterations by performing *in vitro* studies on a primary rat cell-based BBB model. After inhalation, we described a biopersistence mainly in the lungs that can elicit the same kind of mechanism linking persistence of NPs in peripheral tissues and CNS effects^30^. Surprisingly, the serum analysis for pro-inflammatory cytokines and chemokines did not show any evidence of increased inflammatory mediators. While we cannot rule out that it is possible that our techniques failed to detect increased systemic pro-inflammatory factors, it is plausible that other biological circulating mediators, such as lipids or other metabolites^54,55^, may be mediating the response at the BBB and thus warrants further mechanistic investigation.

In regard to neurological processes, chronic neuroinflammation and BBB permeability alteration may lead to neuronal dysfunction and promote neurological deficits. Indeed, these are common phenomenon reported in several CNS pathologies including neurodegenerative diseases^56^. In our studies, quantification of synaptophysin mRNA and protein expression revealed a decrease in the brains of aging NP-exposed animals, suggesting a possible default in synaptic function. Behavioral changes such as increased exploratory behavior, impaired object novelty recognition, and reduced spatial learning have previously been identified as consequences of synaptophysin deletion in knock-out mice^57^. Thus, our results raise the question as to whether the BBB dysfunction and associated neuroinflammation induced after TiO_2_ nano-aerosol exposure may exacerbate neuronal activity decline with aging.

Here, we investigated the impact of TiO_2_ nano-aerosol inhalation in altering BBB integrity and in driving inflammation and neuronal impairment in the brains of young and aged rats. While we did not observe any evidence of brain TiO_2_ NPs translocation, our findings highlight nano-aerosol-exposure mediated BBB dysregulation, neuroinflammation, and impact on neuronal activity under realistic environmental and occupational exposure conditions in both young and aged rats. Dysregulation of BBB structural integrity and permeability were observed in aged exposed rats in contrast with only an alteration in TJ protein (claudin-5), without increased permeability in young exposed rats 28-days post TiO_2_ nano-aerosol-exposure, compared to control animals. Dysregulation of BBB function was also associated with acute neuroinflammation, characterized by a significant increase in cerebral expressions of several pro-inflammatory markers (IL-1β, RANTES, fractalkine, VEGF, IP-10 and IFNγ). In the aging brain, BBB permeability modification and chronic inflammation may be the cause of alterations in neuronal activity, as indicated by decreased synaptophysin mRNA and protein expression. The findings from our study emphasize the need for further research to investigate neurodegenerative process that may occur, as a result of exposure to NPs, in the aging population. Importantly, the hypothesis of direct translocation of TiO_2_ NPs from the nasal mucosa *via* the olfactory bulb appeared unlikely due to the absence of Ti and inflammation in that region after either nasal instillation or inhalation. In consideration of our previously reported findings, this study reveals a potential link between dysregulation of BBB structure and function, and the presence of TiO_2_ NPs in peripheral tissues (lung, spleen, or liver), suggesting systemic-mediated pathways in the observed CNS-related effects. Additional research is needed to identify the mediators responsible for this indirect neuronal effect of TiO_2_ nano-aerosol.

## Material and Methods

### Nanoparticles

TiO_2_ P25 NPs (Aeroxide® P25, 75% anatase 25% rutile, Evonik®) were from Sigma Aldrich (Saint-Quentin Fallavier, France).

### Animals

The animal facilities have full accreditation (D54–547–10) and while conducting the research described in this article, investigators adhered to the guide for care and use of laboratory animals promulgated by the European Parliament and Council (Directive 2010/63/EC, 2010/63/EU, 22 September 2010). The present study was approved by our local ethical committee appointed by the French Ministry of Research and Higher Education (Project n° 00692.01).

Male Fisher F344 rats (from Charles River Laboratories, France), 12–13 weeks old and weighing 300–320 g referred as young adults and 19 months old, weighing 400–425 g referred as aging group^58^, were housed in standard environmental conditions (room humidity 55 ± 10% and temperature 22 ± 2 °C; room under a 12:12 hrs light dark cycle) and maintained with free access to water and A04 diet (Safe diet). The aging rats were fed separately with A04 diet from 1 month of age to 7 months then with A05 diet (Safe diet) adapted to long term studies.

### Generation and monitoring of TiO_2_ nano-aerosol

The inhalation system which has already been described^38^ is mainly composed of an aerosol generation system and inhalation towers for nose-only exposure. The integrated control of the exposure conditions (airflow, temperature and relative humidity) is managed and recorded using dedicated software. Air entering the towers was filtered and conditioned at a temperature of 22 °C ± 2 °C and a humidity of 55 ± 10%. Nanostructured aerosols of TiO_2_ NPs were produced with a rotating brush generator (RBG) (RBG1000 PALAS) using dry nanopowder as the source of NPs. Aerosol concentration was adjusted with feed rate of the RBG operated at constant air flow rate. The aerosol monitoring and characterization were ensured by real time devices (condensation particle counter, electrical low pressure impactor, aerodynamic particle sizer, scanning mobility particle sizer spectrometer, optical light scattering dust monitor) and off-line analyses (gravimetric filter, particle size-distribution by cascade impactor, sampling for TEM observations).

A nano-aerosol is defined as a nano-dispersion with at least one liquid or solid nanophase including nano-objects^59^. In our case, the nano-objects are nanostructured agglomerates composed of TiO_2_ NPs. The morphology and size of TiO_2_ NPs have been previously described in suspension^39^ (spherical particle with primary size of 21.5 ± 7.2 nm; mix of 75% anatase and 25% rutile crystal phases). We recently reported the characteristics of our nano aerosol^30,38^. Briefly, the TiO_2_ nano-aerosol target concentration of 10 mg/m^3^ was achieved consistently: 10.17 ± 3.29 mg/m^3^ for the exposure of adult rats and 10.42 ± 1.80 mg/m^3^ for elderly rats corresponding to 24000 ± 640 particles/cm^3^. Aggregates size distributions demonstrated a count median aerodynamic diameter around 270 nm and a mass median aerodynamic diameter around 905 nm.

### *In vivo* experimental design

Before the beginning of the aerosol exposure period, rats were acclimated to the restraining tubes according to the daily scheme of experiment: 2 periods of 3 hrs exposure. Rats were then exposed to filtered air (controls) or TiO_2_ nano-aerosol (exposed, 10 mg/m^3^) during 2 periods of 3 hrs a day, 5 days/week for 4 weeks. 3 and 28 days after the end of the inhalation exposure period, animals were anesthetized with isoflurane and euthanized. Neuroinflammation and BBB permeability were explored after a recovery period in order to draw appropriate conclusions on the effects of TiO_2_ on the CNS. At the end of the inhalation exposure period, stress due to the experimental protocol might have influence our observations at the CNS level. Organs and blood were collected, sampled, and store at −80 °C until assay.

### Sample preparation and Ti analysis by ICP-MS

Sample preparation was performed as previously described^33,39^. Briefly, total brain tissues were weighed and thawed. Each sample was added with 8 mL of nitric acid + 2 mL of hydrofluoric acid and digested using a Microwave Assisted Reaction System (MARS) Express instrument. The microwave digestion program was 15 min; 150 °C; 1200 W then 15 min; 180 °C; 1200 W. After 20 min cooling, the sample was rinsed 3 times using approximately 20 mL of 2% nitric acid solution in a polytetrafluoroethylene (PTFE) beaker then 2 mL of hydrogen peroxide was added. The beakers were then heated at 180 °C until between 0.1 and 0.5 mL of solution remained. After cooling, the beakers were rinsed 3 times with 2% nitric acid solution before being stored for analysis.

Ti standard solutions for ICP-MS calibration were prepared at concentrations from 2 to 100 ng/L, by diluting a 1 g/L Ti standard stock solution (1.70363.0100, SCP Science) with 2% v/v HNO_3_ and 0.01% v/v Triton X-100. An internal standard solution 25 µg/L of Ge was prepared by diluting a 1000 mg/L stock solution (1.70320.0100, Merck) with 2% v/v HNO_3_. Ti analysis of acidified samples was carried out using a Varian 820-MS. Samples in 2% v/v HNO_3_ were analyzed with the Varian-820-MS. This mineralization method was optimized to obtained assay with 96% recovery and a limit of quantification (LOQ) down to 13.7 ng/g in brain tissue and limit of detection of 4.1ng/g^33^. The LOQ for lung, spleen and liver was about 21.2, 33.1 and 4.7 ng/g, respectively.

### Neuroinflammation assessment using multiplexing approach

The ofactory bulbs were dissected from the rest of the brain (cerebrum + cerebellum), and homogenized separately in 10 volumes of TRIS lysis buffer 50 mM (Sigma) added with proteases inhibitor (Calbiochem) using homogenization for 60 sec in 15 mL TeenPrep Lysing Matrix D (MP Biomedicals). The homogenates were centrifuged for 5 min at 2000 g then the supernatant was recovered for ultracentrifugation (30 min; 26184 g) and stored at −80 °C until assayed.

Multiple cytokines/chemokines (Il1β, IL6, EGF, MIP2, IFNγ, MCP1, IP10, VEGF, Fractalkine, RANTES, TNFα) were analysed in extracts from either the cerebral tissues (cerebrum + cerebellum), olfactory bulbs, or sera using a MILLIPLEX MAP kit (Merck Millipore, Billerica, US) and the Biorad Bioplex-200 analysis instrument (Bio-Rad laboratories, Hercules, US) all according to manufacturer’s instructions. Bioplex manager software was used to generate standard curves and calculate sample cytokines/chemokines concentrations. Assays were performed in duplicate for each brain extract. Results are expressed as mean of duplicate.

### BBB permeability measurement

4 hours before animal necropsy, rats were anesthetized with isoflurane and subcutaneously implanted with mini-osmotic pumps (Alzet model 2001D; DURECT Corp., Cupertino, California). Pumps were filled with atenolol dissolved in PEG200/DMSO (50/50) to deliver at 0.25 mg/kg/h. After 4 hrs, animals were anesthetized with isoflurane and euthanized. Plasma samples and brain were collected and weighed immediately after death. The atenolol concentration was quantified in the two compartments using tandem mass spectrometry coupled with liquid chromatography (LC/MSMS). BBB integrity was estimated by the partition coefficient (Kp) corresponding to the ratio of atenolol brain to plasma concentration (C_brain_ and C_plasma_, respectively).

$$Kp=\frac{{C}_{brain}}{{C}_{plasma}}$$

### LC/MSMS assay for atenolol quantification

Atenolol was used to quantify in brain and plasma as described previously^39^. Brains were mixed in ultrapure water (2 mL/g of tissue) using an Ultraturrax T65 system (IKA-Werke, Staufen, Germany). The extract suspensions (400 µL) were submitted to protein precipitation with 1 mL of methanol previously spiked with internal standard (atenolol-d7 4 µg/mL). After centrifugation (20000 g; 15 min; 4 °C) the supernatant was dried under nitrogen at 40 °C then resuspended in 1 mL 0.75 M NH_4_OH/methanol (80:20 v/v). Plasma (150 µL) was diluted with 150 µL of 0.75 M NH_4_OH/methanol (80:20 v/v) previously spiked with internal standard. Both brain and plasma extracts were submitted to solid-liquid extraction on isolute SLE + columns 1 or 6 mL (Biotage). The two eluates (3 mL of dichloromethane/isopropanol (70:30 v/v) then 3 mL of dichloromethane/isopropanol (70:30 v/v) + 0.2% formic acid) were pooled and evaporated to dryness. The dry extracts were resuspended in 200 µL of 5 mM ammonium acetate/methanol (95:5 v/v). Chromatography was performed using a Shimadzu HPLC system LC 20AD on a Kinetex C18 column (Phenomenex). The total run time was 5 min and the flow rate was 0.4 mL/min. Analyte (20 μL) was injected onto the column placed in an oven at 40 °C.

Detection was done by tandem mass spectrometry (Finnigan TSQ Quantum Discovery with Xcalibur and LC Quan softwares, Thermo) in positive electrospray mode. Tuning parameters were: capillary voltage 3 kV, source temperature 200 °C. The multiple reaction monitoring transitions for atenolol were m/z atenolol 267.18 > 145.1. Analyte was quantified by means of calibration curves using atenolol-d7 as internal standard. For plasma and brain extract assay, calibration ranges were from 1.0 to 200 ng/mL.

### Immunofluorescence

Midsagittal brain sections sampled at 28 days after the end of the inhalation period were embedded in OCT and cut on a cryostat (10 µm) were prepared for either claudin-5 and von Willebrand factor (vWF) double immunofluorescence, or either IL-1β or synaptophysin immunofluorescence. Brain sections were air-dried for 30 minutes and fixed in ice-cold acetone for 30 minutes and then rinsed in PBS. Sections were incubated with 3% BSA (60 min at RT), rinsed in PBS, and incubated with 150 µL per section of the appropriate primary antibody (claudin-5: 1:100, Invitrogen/Life Technologies, Carlbad, CA) and a FITC-tagged vWF (1:1000 dilution, Abcam) or IL-1β (1:1000, Abcam) or synaptophysin (1:1000, Abcam) alone, diluted in rinse wash buffer [1 part 5% blocking solution (0.5 mL Normal Rabbit Serum in 10 mL 3% w/v BSA) and 4 parts PBS] for 1 hr at RT and then rinsed 3 times with PBS. The slides were then incubated in 150 µL per section of the appropriate secondary antibody either Alexa Fluor 555 or Alexa Fluor 488 (1:1000 dilution, Vector Laboratories, Biovalley, Marne la Vallée, France) in the dark for 1 hr at RT. Slides were then rinsed 3 times in PBS and subsequently incubated with Hoescht nuclear stain (1 µL/mL; 150 µL/section) for one minute, rinsed again then cover-slipped with Aqueous Gel Mount (TBS, Fisher Scientific, Waltham, MA). Slides were imaged by fluorescent microscopy at 10x and 40x, using the appropriate excitation/emission filters, digitally recorded, and analyzed by image densitometry using Image J software (NIH). A minimum of 3 locations on each section (2 sections per slide), 3 slides and n = 3 per group were processed/analyzed. IL-1β and synaptophysin were quantified by total amount of fluorescence per unit area (consistent across regions/slides). Claudin-5 (double immunofluorescence, vessel specific) were measured by merging Alexa 488 (fluorescein isothiocyanate) and Alexa 555 (Cy3) signals into Red-Green-Blue (RGB) images. Colocalization was determined by quantifying total fluorescence of overlayed signals. Midbrain analysis included regions of brainstem, hippocampal formation, and cerebral cortex (somatosensory). Forebrain analysis included regions of the cerebral cortex (somatomotor) and cerebral nuclei/caudoputamen/striatum. Only vessels <50 µm were used for claudin-5 analysis.

### Isolation of brain parenchyma

Rat brain capillaries and brain parenchyma were separated as described previously^60,61^. Four to eight rats from each age group were used. Brains were extracted and stored in Hanks balanced salt solution (HBSS) supplemented with 1% (v/v) PSN on ice. The brains were cut sagittally into two halves and the cerebral cortices emptied of white matter. The meninges and the associated vessels were cleaned off by rolling on Whatman 3 mm chromatography paper. The homogenized tissue was pelleted by centrifugation (1500 rpm; 5 min). The pellet was digested in HBSS-1% PSN solution, 1 mg/mL collagenase/dispase, 10 U/μL DNase-I, and 1 μg/mL TLCK for 1 h at 37 °C. Digested tissue was then pelleted by centrifugation (1500 rpm; 5 min; 4 °C). The pellet was resuspended in 20% (w/v) BSA in HBSS-1% PSN solution and centrifuged (2800 rpm; 30 min). The resulting floating white matter corresponding to the brain parenchyma fraction. Centrifugation medium were removed carefully. The remaining parenchyma fraction was resuspended in centrifugation medium then half-dissolved in HBSS-1% PSN solution and pelleted by centrifugation (1500 rpm; 15 min). The brain parenchyma pellet was then washed several times before storage at −80 °C.

### Transcription profiling

RNA was isolated from brain parenchyma fraction using the RNeasy Mini kit (Qiagen, France), according to the manufacturer’s instructions. The concentration and purity of the RNA samples were assessed spectrophotometrically at 260 and 280 nm using the NanoDrop ND-1000 instrument (NanoDrop Technologies, Wilmington, DE, USA). The A260/280 ratio ranged between 1.8 and 2. A sample of 0.5 µg of total RNA was converted to cDNA with random primers in a total of 10 µL using an RT2 first stand kit (Qiagen, France). The cDNA was diluted with DNA/RNAse-free distilled water to a volume of 110 µL.

The quantitative expression of synaptophysin was determined using 0.4 µM cDNA for each primer set in the RT2 Pathway Focus profiler array from Qiagen. The RT2 Profiler array consists of a previously validated qRT-PCR primer set (1 µL) for synaptophysin. Thermocycling was carried out in a CFX96 real-time PCR detection system (Bio-Rad) using SYBR green fluorescence detection. The final reaction mixture contained 2 µL of diluted cDNA, 1 µL of one of the specific primer, 12.5 µL of distilled water and 9.5 µL of SYBR green master mix. The specific amplification conditions were 2 min at 50 °C, 10 min at 95 °C followed by 40 amplification cycles at 95 °C for 0.5 min, and 60 °C for 1 min to reinitialize the cycle again. The specificity of each reaction was also assessed by melting curve analysis to ensure the presence of only one product. Relative gene expression values were calculated as 2^−ΔCT^, where ΔCT is the difference between the cycle theshold (CT) values for genes of interest and housekeeping genes (hypoxanthine guanine phosphoryltransferase or Hprt).

### Statistical analysis

For brain content Ti analysis, statistical analysis was performed using Stata 14 (StataCorp LP,TX,USA). After Box cox transformation on Ti concentration variable, statistical comparisons were accomplished using two-way ANOVA (the two determinants being treatment and time) followed by Bonferroni post-hoc test. After homogeneity of variance confirmation by Hartley or Bartlett test, atenolol partition coefficients were analyzed using one-way ANOVA between control and exposed groups followed by Tukey post hoc test (Prism 5.1 program, GraphPad Software, Inc, San Diego CA). Cytokines and chemokines assays were analyzed either using one-way ANOVA followed by Tukey post hoc test when Hartley or Bartlett test confirmed homogeneity of variances (Il1β, IFNγ, VEGF and IP10 for both aged groups; RANTES for aging group) or Kruskal-Wallis test followed by test by Dunn’s post hoc test (RANTES for young adult groups). Immunofluorescence endpoints were analyzed using a one-way ANOVA between treatment groups followed by Holm-Sidak post hoc test (SigmaPlot SyStat Software Inc, San Joes, CA) and data expressed as mean ± SEM. Finally, mRNA expressions of synaptophysin were analysed using two-tailed Student’s t-test after Hartley test confirmed homogeneity of variances. All changes were considered statistically significant at p < 0.05.
